# Hypovitaminosis D: a novel finding in primary ciliary dyskinesia

**DOI:** 10.1186/s13052-015-0119-5

**Published:** 2015-02-22

**Authors:** Virginia Mirra, Carlo Caffarelli, Marco Maglione, Rossella Valentino, Giuseppe Perruolo, Claudia Mazzarella, Laida Lisa Di Micco, Silvia Montella, Francesca Santamaria

**Affiliations:** Department of Translational Medical Sciences, Federico II University, Via Sergio Pansini, 5-80131 Naples, Italy; Department of Pediatrics, Department of Clinical and Experimental Medicine, Azienda Ospedaliera-Universitaria, University of Parma, Parma, Italy; National Council of Research, Institute of Experimental Endocrinology and Oncology, Naples, Italy

**Keywords:** Primary ciliary dyskinesia, Vitamin D, Quality of life

## Abstract

**Background:**

A relationship between low levels of serum vitamin D and respiratory infections has been established. No study has examined the frequency and clinical relevance of vitamin D deficiency in patients with primary ciliary dyskinesia (PCD).

**Methods:**

Vitamin D levels were measured in 22 PCD patients (7 females, 10.5 years, range, 2–34 years). In PCD, pulmonary function tests (PFTs), sputum microbiology, self-reported physical activity (PA) level, and quality of life (QoL) by means of the Saint George’s Respiratory Questionnaire (SGRQ), were also assessed.

**Results:**

Seventy-two percent of PCD patients were vitamin-D deficient-to-insufficient and 28% were sufficient. No differences in PFTs parameters were found between vitamin D deficiency-to-insufficiency and sufficiency groups. Patients with vitamin D deficiency-to-insufficiency had significantly higher SGRQ total scores, and thus poorer QoL (*p* = 0.03). Seventy-nine percent of PCD subjects had limitations in performing vigorous activities, and 53% performed less than 3 hours of PA per week. Vitamin D deficiency-to-insufficiency and sufficiency groups did not show any differences in age at PCD diagnosis or at onset of respiratory symptoms, BMI, atopy, current asthma or bronchiectasis. However, 79% of patients with bronchiectasis had vitamin D deficiency-to-insufficiency. No differences were found in the rate of positive sputum cultures and in the number of antibiotic courses between the two groups.

**Conclusions:**

Hypovitaminosis D is common in PCD patients, and is associated with poorer QoL. We recommend the assessment and treatment of hypovitaminosis D to be included in the routine management of PCD.

## Introduction

Primary ciliary dyskinesia (PCD), a genetic disorder of cilia function and ultrastructure with *situs viscerum inversus* occurring in 50% of patients, is characterized by impaired mucociliary clearance and recurrent-to-chronic respiratory infections [1]. Early presenting symptoms include neonatal respiratory distress and upper airway disease. Lower airways are commonly involved in PCD, and recurrent pneumonia, chronic asthma-like symptoms, and bronchiectasis are the hallmarks of the disease [2]. As a consequence, pulmonary function becomes progressively impaired and respiratory failure may eventually occur [3,4].

Although vitamin D plays a major role in bone health, recent evidence suggests that low levels may contribute to several chronic diseases [5,6]. A number of studies indicate that individuals with lower vitamin D are at higher risk of respiratory infections [7-10]. Vitamin D deficiency is common also in adults with non-cystic fibrosis (CF) bronchiectasis, a condition characterized by a vicious circle of airway inflammation and infection [11].

In PCD, failure of mucus clearance system due to defective ciliary function results in reduced airway defense against bacteria [1]. To our knowledge, the links between vitamin D and PCD lung disease have never been investigated. We hypothesize that, likewise other chronic respiratory disorders, patients with PCD may have hypovitaminosis D. Therefore, we measured the total circulating levels of 25-hydroxy cholecalciferol [25(OH)D] in children, adolescents and adults with stable PCD lung disease. Moreover, we explored whether 25(OH)D concentrations were associated with pulmonary function parameters, sputum culture, patients’ quality of life (QoL), and self-reported physical activity (PA) level.

## Methods

### Patient population

We conducted a prospective, cross-sectional study of 22 consecutive PCD subjects (median age, 10.5 years; range, 2–34 years; 7 adults; 7 females) attending the Department of Translational Medical Sciences, Federico II University, Naples, Italy, the reference center for PCD in Campania, Southern Italy. Patients lived in Naples metropolitan area (latitude, 40° 49’ N; elevation, 17 m) and were evaluated from March through June 2012. Diagnosis of PCD was made at a median age of 5.9 years (range, 0.1-27) and was based on the demonstration of abnormal motility and ultrastructural defects of cilia. Eighty-two percent of patients (18/22) had *situs viscerum inversus*, nobody had heterotaxy. Table 1 summarizes the characteristics of the study population. Thirty-two percent of cases had atopy that was diagnosed on the basis of the results of skin prick tests. Current asthma occurred in 36% of patients, as assessed by standardized questionnaires [12,13]. Sixty-four percent of cases (14/22) had bronchiectasis at chest high resolution computed tomography performed in stable conditions at least in the previous 6 months for assessing disease severity at some time point during follow-up. Inclusion criteria were a confirmed diagnosis of PCD, and PCD lung disease stability was defined as previously reported [14]. Exclusion criteria were: airway infections or asthma exacerbations during the 4 weeks prior to enrollment; current smoking; long term use of oral steroids at any dose; antibiotic treatment in the last 4 weeks before enrollment; prescription of over-the-counter calcium or vitamin-D supplements prior to, or during the study period. None of the subjects had any neoplastic, metabolic, hepatic, and cardiovascular or other concurrent medical disorders (i.e., renal or malabsorptive diseases). All participants reported neither being current smokers nor having been exposed to smoke in the previous 4 weeks.

**Table 1:** **Characteristics of patients with primary ciliary dyskinesia (n = 22)**

***Clinical data***	
Age (yr)	10.5 [2-34]^a^
Gender (F/M)	7/15
*Situs viscerum inversus*, n (%)	18 (82)
BMI, kg/m^2^	18.5 [13-37]^a^
Age to diagnosis, yrs	6 (0.1-27)^a^
Age at onset of respiratory symptoms, yrs	0.08 (0–9.5)^a^
Atopy, n (%)^b^	7 [32]
Current asthma, n (%)	8 [36]
Bronchiectasis at high resolution computed tomography, n (%)	14 (64)
***Cilia ultrastructural defects, n (%)***	
Outer or combined outer and inner dynein arms absence	12 (77)
Isolated inner dynein arm absence	2 [9]
Isolated axonemal disorganization	1 [5]
Axonemal disorganization and inner dynein arm absence	2 [9]

^a^Median and range values.

^b^Defined on the basis of results of skin testing to the most common seasonal and perennial local allergens.

On the study day, in the morning, patients underwent serum vitamin D levels measurement, pulmonary function tests (PFTs), deep throat or sputum culture, and completed health-related QoL and self-reported PA questionnaires. The procedures were in accordance with the Helsinki Declaration guidelines on human experimentation. The study was conducted without any support from the pharmaceutical industry, after approval by the local institutional review board. Subjects or their legal guardians gave informed written consent after extensive information about the study procedures.

### Vitamin D measurement

A single determination of vitamin D levels, measured as total 25(OH)D, was performed on blood samples obtained between 08:00 a.m. and 09:00 a.m. after overnight fast, using the chemiluminescent method (Liasion, DiaSorin, Saluggia, Italy) [15]. Vitamin D levels were categorized as being sufficient when >30 ng/ml (>75 nmol/L), insufficient between 20 and 30 ng/ml (50 and 75 nmol/L), and deficient when <20 ng/ml (<50 nmol/L) [6].

### Pulmonary function testing

Cooperating PCD subjects underwent PFTs (MasterScreen®Body, VIASYS Healthcare GmbH, Wuerzburg, Germany). Forced vital capacity (FVC), forced expiratory volume in 1 second (FEV_1_), forced expiratory flow between 25% and 75% of FVC (FEF_25–75_), functional residual capacity (FRC), and residual volume (RV) were expressed as % of predicted, while FEV_1_/FVC ratio was expressed as % [16].

### Quality of life and self-reported physical activity assessment

In order to assess health-related QoL, 19/22 patients with PCD (86%) aged ≥6 years completed the St. Georges Respiratory Questionnaire (SGRQ; 17), a disease-specific measure developed for asthma and chronic obstructive pulmonary disease (COPD) that has been validated also in children and adults with PCD [18,19]. SGRQ score ranges from 0 to 100 (100 indicating the maximum impairment). The youngest children were helped by their parents in answering the SGRQ questions. We also administered a previously published questionnaire to the same 19 patients for assessing their self-reported PA [20].

### Microbiological evaluation

Based on cultures results, we defined chronic bacterial colonization as persistence of specific bacteria for at least 6 months, with at least 3 positive cultures [4]. For each subject we also recorded the number of antibiotic courses performed in the past 12 months. All investigators were blinded to the other results.

### Statistical analysis

Data are presented as median and range. Mann–Whitney *U* test and Fisher’s test assessed comparisons among variables. A *p* value <0.05 was considered statistically significant. Data were analyzed with a statistical software package (SPSS-PC, version 13.0; SPSS; Chicago, IL).

## Results

**Figure 1:**
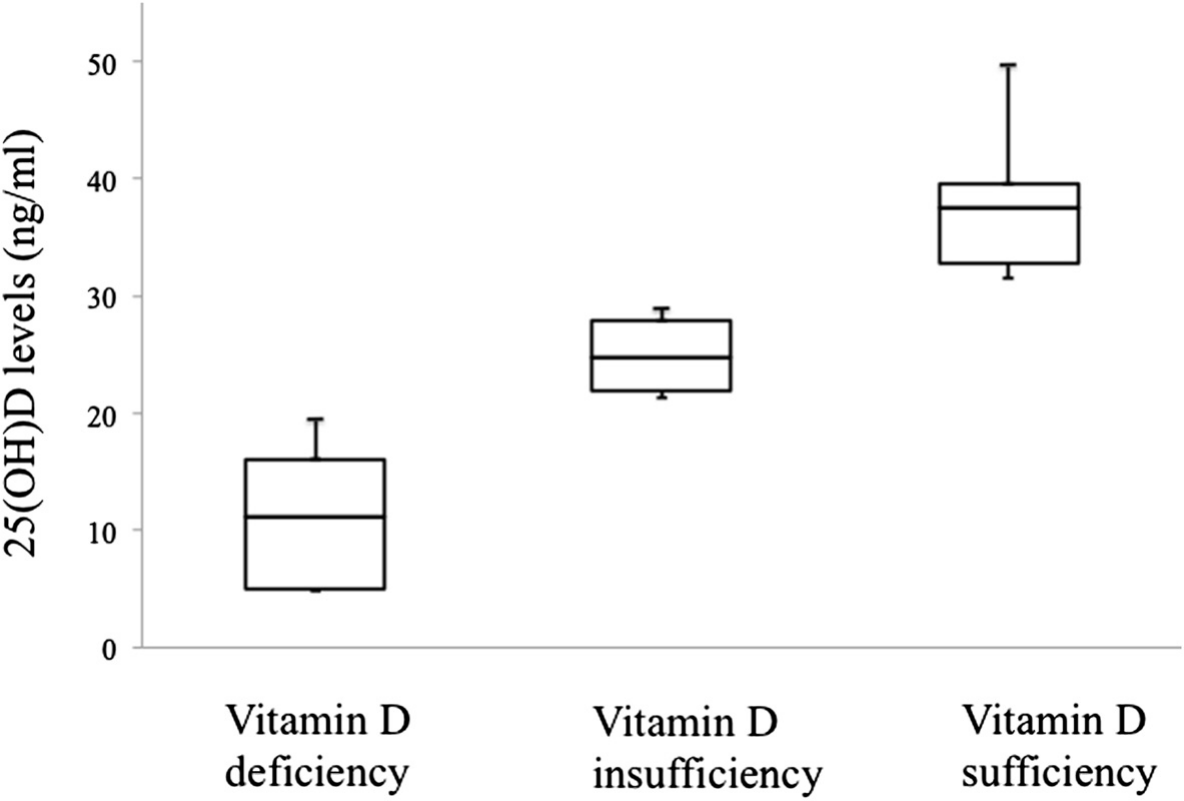
**Serum 25(OH)D levels in PCD patients with vitamin D deficiency, insufficiency and sufficiency.**

Median serum 25(OH)D levels in PCD were 25 ng/mL (4.8-49). Seventy-two percent of patients had vitamin D deficiency-to-insufficiency, with 4/22 cases (18%) exhibiting 25(OH)D levels <20 ng/ml and 12/22 patients (54%) having 20–30 ng/ml, while 6/22 cases (28%) had >30 ng/ml. Figure 1 illustrates the serum 25(OH)D levels in PCD patients with vitamin D deficiency, insufficiency and sufficiency.

Twelve cooperative PCD subjects underwent PFTs. Median FVC, FEV_1_, and FEF_25–75_ were 97% (58–134), 86% (40–111), and 49% (16–100) predicted, respectively, while the FEV_1_/FVC ratio was 73% (58–85). Median FRC and RV were 87% (46–173) and 74% (18–275) predicted, respectively. No significant differences in PFTs were found between vitamin D deficiency-to-insufficiency and vitamin D sufficiency groups (*p* >0.05).

Total SGRQ score was 19 (9–65). PCD patients with vitamin D deficiency-to-insufficiency had significantly higher total scores at SGRQ (20 *versus* 17, *p* = 0.03) than those with vitamin D sufficiency. In the responses to the questions on PA, 10% of patients reported being moderately-to-highly limited, 26% slightly limited, and 63% not limited at all by respiratory symptoms in everyday-life activities. Fifty-two percent of cases reported moderate-to-severe limitations in performing vigorous activities, while 26% had only slight difficulties, and 21% denied any difficulty at all. Fifty-three percent of patients performed less than 3 hours of physical training every week, while only 5% spent more than 7 hours. Respiratory symptoms limited everyday-life activities at least slightly in 38% and 33% of patients with vitamin D deficiency-to-insufficiency or sufficiency, respectively (*p* = 1). Vigorous activity appeared at least slightly limited in 77% and 83% of the cases with vitamin D deficiency-to-insufficiency or sufficiency, respectively (*p* = 1). Fifty-four percent of subjects with vitamin D deficiency-to-insufficiency and 33% of patients with sufficiency reported performing > 2 hours per week of physical training (*p* = 0.6).

Vitamin D deficiency-to-insufficiency and sufficiency groups did not show any significant differences in BMI (19 *versus* 17 Kg/m^2^, *p* = 0.7) and in the ages at PCD diagnosis (8.3 *versus* 2.3 years, *p* = 0.4) or at the onset of respiratory symptoms (13.5 *versus* 6.8 years, *p* = 0.7). No significant difference was found in vitamin D levels from atopic and non atopic patients (21.9 *versus* 27.9 ng/ml, *p* = 0.5), from asthmatic and non asthmatic subjects (24.2 *versus* 26.3 ng/ml, *p =* 0.4), or from patients with or without bronchiectasis (24.5 *versus* 30.4 ng/ml, *p* = 0.6). Within the bronchiectasis group, 11 out of 14 cases (79%) had vitamin D deficiency-to-insufficiency and 3 patients (21%) had vitamin D sufficiency, while among the 8 cases with no bronchiectasis 5 (62%) had vitamin D deficiency-to-insufficiency and 3 (38%) had vitamin D sufficiency (*p =* 0.6).

Deep throat or sputum cultures were negative in 13/22 subjects (59%). The most frequently isolated pathogens were *Haemophilus influenzae* (27%), *Staphylococcus aureus* (14%), *Streptococcus pneumoniae* (9%), *Klebsiella spp* (9%), and *Pseudomonas aeruginosa* (4%). No significant difference in the rate of positive cultures was found between the vitamin D deficiency-to-insufficiency and the sufficiency groups (68% *versus* 50%, *p* = 0.6). Only 2 patients with vitamin D insufficiency met the criteria for chronic colonization by *Haemophilus influenzae*. No patients were chronically colonized by *P. aeruginosa*.

During the 12 months preceding the study, patients had undergone a median of 4 antibiotic courses (range, 0–7), but no difference was found between vitamin D deficient-to-insufficient and sufficient subjects (3.5 *versus* 4 courses, *p* = 0.9).

## Discussion

To our knowledge, vitamin D status has never been investigated in PCD. In this pilot, cross-sectional study, only 28% of PCD patients living at a latitude of 40°52’ N had sufficient 25(OH)D serum levels during spring. Our findings show that more than two thirds of PCD children have hypovitaminosis D, which is associated with worse quality of life. However, we could not find any significant relation between PCD-associated vitamin D status and pulmonary function, sputum microbiology, past exacerbations, atopy, or current asthma.

In addition to latitude and season, factors affecting vitamin D status include skin pigmentation, sun-related behavior, obesity, vitamin D dietary intake and outdoor/indoor activities, or also reduced ultraviolet B (UVB) radiations due to atmospheric pollution [5,6,21-23]. Our study was not designed to investigate the reasons for low levels of serum vitamin D. However, in our population BMI excluded obesity, but self reported PA indicated that patients were quite sedentary. A high proportion of cases (79%) had limitations in performing vigorous activities, and approximately 50% spent less than 3 hours per week doing PA, thus suggesting that PCD likely makes patients inactive [20,24].

PCD leads to chronic respiratory symptoms and loss of lung function, with great impact on health and significant restriction of life-style [25,26]. The association of vitamin D levels with quality of life in PCD patients may be explained by noncalcemic effects of vitamin D [6]. Vitamin D has an antimicrobial activity especially against airway pathogens. In bronchial epithelial cells, vitamin D increases the expression of cathelicidins which may prevent bacterial infections [27]. Cord blood levels of 25(OH)D have a strong inverse association with early life airways infections [8]. Vitamin D supplementation significantly reduced the risk of influenza A among Japanese schoolchildren [7] and of winter infections in vitamin D deficient Mongolian children [9]. Of interest is that the need for antibiotics decreases in older adults treated with oral vitamin D [10]. Finally, the resolution of inflammatory responses during tuberculosis treatment is accelerated by the addition of vitamin D [28]. Also chronic lung disorders have been associated to vitamin D status. Increased incidence of asthma and vitamin D deficiency appear related, and the decreased defense against airway pathogens triggering childhood and adulthood wheezing primarily accounts for this [29,30]. Moreover, reduced asthma control, lung function impairment and airway remodeling significantly correlate with hypovitaminosis D [31-33]. Vitamin D deficiency and chronic obstructive pulmonary disease (COPD) appear strongly associated, likely because of subjects’ physical impairment and reduced PA, patients’ old age which is frequently associated with low vitamin levels, continuous steroid treatment inducing vitamin D catabolism, and malnutrition [34]. Vitamin D deficiency is common in adult non-CF/non-PCD bronchiectasis and correlates with disease severity, although the mechanism is unclear [11]. Likewise non-CF bronchiectasis, the recurrent-to-chronic infectious nature of PCD pulmonary disease might be associated to vitamin D status, and this prompted us to investigate on it. Of interest is that hypovitaminosis D is almost universal in CF, due to a combination of inadequate absorption, impaired metabolism, and lack of sun exposure [35]. Chest sepsis, albeit milder in PCD than CF, is a feature of both disorders [36]. However, vitamin D malabsorption is not seen in PCD, and hence, the pathogenesis of hypovitaminosis D in PCD can be somewhat different.

Markedly reduced nasal nitric oxide (nNO) is typical of PCD [37,38]. Explanations include NO trapping and subsequent breakdown in the airways due to mucus accumulation or paranasal sinuses agenesis [39,40], or also reduced nNO synthesis due to decreased expression, or abnormal activity of nitric oxide synthase (NOS) isoenzymes [40]. Regulation of NOS potentially contributes to the antimicrobial effects of vitamin D, but conflicting data were reported, ranging from a 1,25(OH)2D3-mediated induction of NOS expression in a human macrophage-like cell line to inhibitory actions of 1,25(OH)2D3 on NOS [41,42]. These findings indicate an association between vitamin D and NO production [43], and it is tempting to suppose that the NO-vitamin D relationship plays a role also in the PCD airways host defence.

This study has some limitations. The small sample size from a single centre could affect generalizability and, perhaps, the absence of significant differences among groups. However, the condition is rare, and this, combined with the criteria of stable disease, likely restricted patients’ inclusion. We did not compare stable *versus* unstable patients for determining the potential of exacerbations and/or the effect of antibiotics on vitamin D status. The cross-sectional nature of the study did not allow to evaluate vitamin D status longitudinally, particularly after adequate supplementation. We did not assess the patients’ daily dietary vitamin D intake, and finally, we did not measure vitamin D-binding protein, a serum protein with immunomodulatory functions that, as well as vitamin D, could be relevant in PCD [44]. Notwithstanding these drawbacks, our study provides the novel valuable information that a high proportion of PCD subjects have vitamin D deficiency-to-insufficiency, with worse QoL than sufficient patients. Determination of vitamin D levels in the early phases of PCD might also clarify the mechanism underlying the association between the two events, including whether the former precedes the latter. In COPD, osteoporosis also due to abnormal vitamin D status increases morbidity [45]. Surprisingly, osteoporosis was never investigated in PCD. Hopefully, following our novel information, a study might be promoted for preventing or treating potential PCD-associated osteoporosis.

In conclusion, our findings show that stable PCD children and adults commonly have hypovitaminosis D, with poorer quality of life than those without. This suggests that assessment of serum vitamin D levels might be included in the management of PCD patients. However, larger studies are warranted to clarify the relationship between hypovitaminosis D and PCD lung disease.

## Abbreviations

COPD: Chronic obstructive pulmonary disease
CF: Cystic fibrosis
FVC: Forced vital capacity
FEV_1_: Forced expiratory volume in 1 second
FEF_25–75_: Forced expiratory flow between 25% and 75% of FVC
FRC: Functional residual capacity
nNO: Nasal nitric oxide
NOS: Nitric oxide synthase
PCD: Primary ciliary dyskinesia
PFTs: Pulmonary function tests
QoL: Quality of life
RV: Residual volume
SGRQ: Saint George’s Respiratory Questionnaire
PA: Self-reported physical activity
SPSS-PC: Statistical software package
UVB: Ultraviolet B
